# An Age of Specialism

**Published:** 1921-06-18

**Authors:** 


### ON COMPARATIVE MEDICINE.

### An Age of Specialism.

We live in an age of specialism, largely born of utilitarianism, and while a judicious mixture of both is very desirable, one must not lose sight of the fact that too much of one or both leads, in a profession such as medicine, to a very narrow outlook, and prevents the victim from taking that broad view of life which is the only sure guide even to the specialist. There are those who favour the shortening of the medical curriculum by the curtailment of the time which the medical student has to spend on the "preliminary sciences."

But the problems confronting the medical man in the human being differ only in degree from those presented in the amœba, and there is this advantage in studying the latter, that the problems are presented in a much simpler form, and when mastered lay the foundations of human physiology. It is only by a progressive study of biology, beginning with the lowest forms of plant and animal life, that one comes to realise the eternal struggle of life against surroundings which are leagued together to destroy it, and having realised this to apply that knowledge to the ceaseless struggle of human beings against disease and death.

How many medical men, especially of the younger generation, have read through that classic of, one might almost say, comparative medicine—viz., the "Origin of Species"? Very few, we venture to say, and we would strongly advise those who have not to do so at once. There they will find a full vindication of the need to study biology in its broader aspects. Darwin, in building up his theory of evolution, cites examples from every living thing, whether in the plant or animal world, shows incidentally how interdependent living beings are, and how one can draw no conclusion from an exclusive study of any one species or genus, no matter how detailed it may be. The work of Pasteur on plants, insects, and animals is another classical example of the advantages of comparative medicine in the elucidation of the problems of human disease.

We cannot think, therefore, that the desire for curtailment of the study of general biology is going to bring any benefit to the medical student, but, on the contrary, feel that it is going to narrow his outlook, and will, in the long run, rear a race of medical men who are incapable of tackling any of the higher problems of medicine. They may have no ideas above the prescribing of drugs, a form of treatment which has kept the medical profession down too long. Even dentists have found that too narrow and technical a training has been to their detriment, and are now being taught physiology, pathology, and anatomy beyond their immediate needs—some even taking additional qualifications in general medicine. After all, there is no better motto for medicine than the saying of Terence: "Homo sum; humani nihil a me alienum puto."

